# Large concomitant outbreaks of acute gastroenteritis emergency visits in adults and food-borne events suspected to be linked to raw shellfish, France, December 2019 to January 2020

**DOI:** 10.2807/1560-7917.ES.2020.25.7.2000060

**Published:** 2020-02-20

**Authors:** Anne Fouillet, Nelly Fournet, Cécile Forgeot, Gabrielle Jones, Alexandra Septfons, Léa Franconeri, Katia Ambert-Balay, Jeannot Schmidt, Patrick Guérin, Henriette de Valk, Céline Caserio-Schönemann

**Affiliations:** 1 Santé publique France, Data Science Division, Saint-Maurice, France; 2 Santé publique France, Infectious Diseases Division, Saint-Maurice, France; 3 National Reference Centre for Gastroenteritis Viruses, Laboratory of Biology and Pathology, Centre Hospitalier Universitaire (CHU) of Dijon, Dijon, France; 4 University Bourgogne Franche-Comté, AgroSup Dijon, PAM UMR A 02.102, Dijon, France; 5 Emergency department, CHU of Clermont-Ferrand and Centre Hospitalier (CH) of Riom, Clermont-Ferrand, France; 6 SOS Médecins Nantes, Nantes, France

**Keywords:** gastrointestinal diseases, foodborne diseases, syndromic surveillance, mandatory reporting, shellfish, norovirus, emergency

## Abstract

On 27 December 2019, the French Public Health Agency identified a large increase in the number of acute gastroenteritis and vomiting visits, both in emergency departments and in emergency general practitioners’ associations providing house-calls. In parallel, on 26 and 27 December, an unusual number of food-borne events suspected to be linked to the consumption of raw shellfish were reported through the mandatory reporting surveillance system. This paper describes these concomitant outbreaks and the investigations’ results.

On 27 December 2019, Santé publique France, the French Public Health Agency (SpFrance), identified through its syndromic surveillance system (SurSaUD) [1], a large increase in the number of visits for gastrointestinal diseases, primarily acute gastroenteritis (AGE) and vomiting recorded for 26 December 2019. This was seen both in emergency departments (ED) of the Organisation de la surveillance coordonnée des urgences (OSCOUR) network and in the emergency general practitioners’ (GP) associations that provide house-calls, SOS Médecins. In parallel, on 26 and 27 December, an unusually high number of food-borne event outbreaks (FEO) suspected to be linked to the consumption of raw shellfish were reported to SpFrance through the mandatory reporting surveillance system. Here we describe these concomitant outbreaks and the investigations’ results.

## Gastroenteritis outbreak alert

The number of visits for AGE and vomiting for both ED and emergency GP house-call associations increased drastically on 26 and 27 December (n = 3,925 and 4,896 respectively) in mainland France and Corsica for people 15 years of age and over, compared with the mean daily number of visits from 1 to 25 December 2019 (n = 1,161).

In parallel, on 26 and 27 December, 43 FEO suspected to be linked to the consumption of raw shellfish, primarily oysters, were reported to SpFrance through the mandatory reporting surveillance system. This number was unusually high compared with previous years. Since 2006, between 3 and 22 FEO linked to the consumption of raw shellfish have been reported per year in December.

The French Ministry of Health (DGS) was notified on 27 December 2019 of the increased number of consultations for AGE observed through the syndromic surveillance system, as well as the unusual number of FEO reporting with a suspected link to raw shellfish.

## Syndromic surveillance system

In 2004, SpFrance implemented the syndromic surveillance system SurSaUD based on two morbidity data sources: the OSCOUR network and SOS Médecins [1,2]. The system allows for monitoring of long-term disease trends and provides early warning of seasonal or unexpected outbreaks [3-5]. At present, the system collects daily individual data, administrative and demographic information and coded medical diagnosis, from 700 ED (92.3% of the national ED attendances) and from 62 of 63 SOS Médecins associations covering all of France, including overseas territories.

## Food-borne event outbreaks mandatory reporting surveillance system

FEO are defined as the occurrence of two or more cases of a similar illness, usually gastroenteritis, following the ingestion of a common food. Notification of FEO is mandatory in France since 1987. FEO are first reported to either the regional health agency (ARS) or the district service (DDecPP) of the French Directorate General for Food (DGAL), or to both. They are then reported at the national level to SpFrance and the DGAL at the Ministry of Agriculture.

## Description of the outbreak

**Figure 1:**
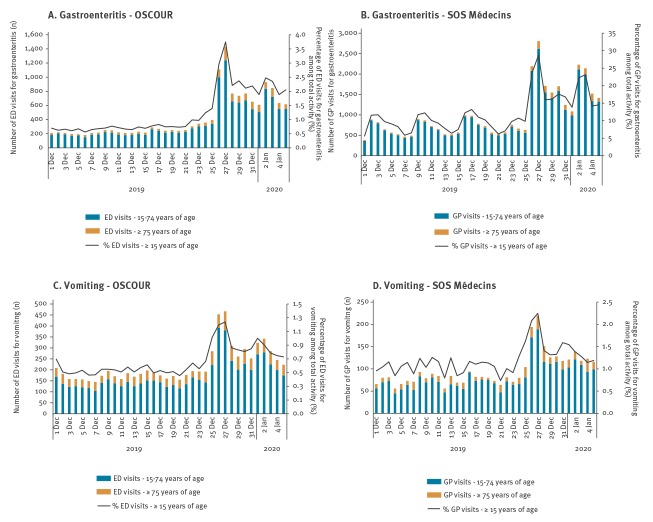
Daily number of ED (OSCOUR) and SOS Médecins visits for acute gastroenteritis (A, B) and vomiting (C, D), and proportion of the total activity, for people 15 years of age and over, 1 December 2019–5 January 2020, mainland France and Corsica

ED: emergency department; GP: general practitioner; OSCOUR: Organisation de la surveillance coordonnée des urgences; SOS Médecins: emergency GP house-call association.

The outbreak period was defined as 26 December 2019 to 5 January 2020. Among the five gastrointestinal indicators monitored daily by the system (AGE, vomiting, diarrhoea, abdominal pain and food poisoning), only AGE and vomiting showed a major increase during the outbreak period (Figure 1).

The number of visits for AGE and vomiting for both ED and emergency GP house-call associations (n = 33,500) in mainland France and Corsica, from 26 December 2019 to 5 January 2020 was above the expected number of 19,750, the mean proportion calculated for the same period of the previous 7 years (2012–2018) applied to the total activity during the outbreak period.

**Figure 2:**
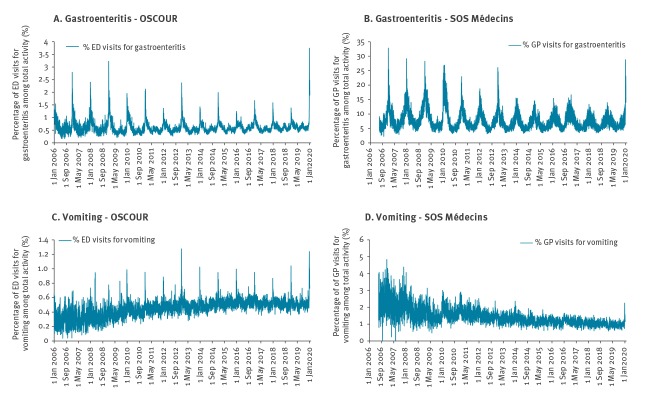
Daily proportion of ED (OSCOUR) and SOS Médecins visits for acute gastroenteritis (A, B) and vomiting (C, D) for people 15 years of age and over, 1 January 2006–5 January 2020, mainland France and Corsica

ED: emergency department; GP: general practitioner; OSCOUR: Organisation de la surveillance coordonnée des urgences; SOS Médecins: emergency GP house-call association.

For people 15 years of age and over, there were 8,957 registered ED attendances for AGE and 3,386 for vomiting, representing 2.4% and 0.9% of the total ED coded activity among adults, respectively (Table). An increase in activity for AGE and vomiting is typically observed during the end-of-year holiday period, however, the observed AGE proportion was the highest registered since the beginning of syndromic surveillance and that for vomiting was the second highest over the same period (Figure 2). During the outbreak period, the proportions of ED attendances for AGE and vomiting among the total activity were, respectively, 1.9 and 1.4 times higher than the mean proportions calculated over the same period of the previous 7 years (2012–2018) (Table). During this period, at least 50% of the approximately 700 ED in France and 85% of SOS Médecins house-call associations were captured by the system.

**Table:** Number, proportion of ED (OSCOUR) and SOS Médecins visits, and ratio for acute gastroenteritis and vomiting among the total activity by age group, 26 December 2019–5 January 2020, mainland France and Corsica (n = 33,500)

Place of visit	Age group (years)	Total activity (number of visits)	Acute gastroenteritis				Vomiting			
			Number of visits	Observed proportion (O)	Expected proportion (E)	Ratio (O/E)	Number of visits	Observed proportion (O)	Expected proportion (E)	Ratio (O/E)
**Outbreak period from 26 December 2019 to 5 January 2020**										
ED	< 15	107,396	6,765	6.3	6.3	1.0	2,246	2.1	1.8	1.1
	15–74	298,965	7,777	2.6	1.4	1.9	2,723	0.9	0.6	1.4
	≥ 75	72,240	1,180	1.6	0.9	1.9	663	0.9	0.7	1.3
	**Total ≥** **15 years of age**	**371,205**	**8,957**	**2.4**	**1.3**	**1.9**	**3,386**	**0.9**	**0.6**	**1.4**
SOS Médecins	< 15	39,562	4,958	12.5	9.4	1.3	931	2.4	2.0	1.2
	15–74	89,776	18,117	20.2	12.1	1.7	1,327	1.5	1.2	1.2
	≥ 75	14,214	1,485	10.4	5.3	2	226	1.6	1.1	1.5
	**Total ≥** **15 years of age**	**103,990**	**19,602**	**18.8**	**11.0**	**1.7**	**1,553**	**1.5**	**1.2**	**1.2**
**First peak (27 December 2019)**										
ED	< 15	10,447	567	5.4	6.2	0.9	191	1.8	1.8	1
	15–74	29,952	1,213	4	1.8	2.2	379	1.3	0.7	1.7
	≥ 75	7,597	193	2.5	1.1	2.3	86	1.1	0.7	1.6
	**Total ≥** **15 years of age**	**37,549**	**1,406**	**3.7**	**1.7**	**2.3**	**465**	**1.2**	**0.7**	**1.7**
SOS Médecins	< 15	3,192	328	10.3	8.6	1.2	65	2	1.8	1.1
	15–74	8,509	2,619	30.8	17.1	1.8	189	2.2	1.5	1.5
	≥ 75	1,239	187	15.1	7.0	2.2	30	2.4	1.2	2
	**Total ≥** **15 years of age**	**9,748**	**2,806**	**28.8**	**15.5**	**1.9**	**219**	**2.2**	**1.5**	**1.5**
**Second peak (2 January 2020)**										
ED	< 15	9,499	674	7.1	6.4	1.1	193	2.0	1.9	1.1
	15–74	29,592	821	2.8	1.4	1.9	272	0.9	0.6	1.5
	≥ 75	7,314	93	1.3	0.9	1.5	62	0.8	0.6	1.3
	**Total ≥** **15 years of age**	**36,906**	**914**	**2.5**	**1.3**	**1.9**	**334**	**0.9**	**0.6**	**1.4**
SOS Médecins	< 15	2,812	464	16.5	10.5	1.6	84	3	2.1	1.4
	15–74	8,801	2,116	24	13.3	1.8	121	1.4	1.2	1.1
	≥ 75	1,225	113	9.2	4.6	2	18	1.5	1.1	1.3
	**Total ≥** **15 years of age**	**10,026**	**2,229**	**22.2**	**11.9**	**1.9**	**139**	**1.4**	**1.2**	**1.2**

E: mean proportion of visits among the total activity of the seven previous periods (2012–2018); ED: emergency department; O: observed proportion of visits among the total activity; OSCOUR: Organisation de la surveillance coordonnée des urgences; SOS Médecins: emergency general practitioner house-call association.

For the SOS Médecins house-call associations, 19,602 AGE visits and 1,553 visits for vomiting were registered for people 15 years of age and over, representing 18.8% and 1.5% of the total activity, respectively (Table). These proportions were 1.7 and 1.2 times higher than the mean proportions calculated over the same period of the previous 7 years (2012–2018). The observed AGE proportions was the highest registered since the past 7 years and that for vomiting was the second highest over the same period (Figure 2).

Two peaks in activity were observed on 27 December 2019 and 2 January 2020 (Figure 1). The increased activity for AGE and vomiting predominantly impacted people aged 15–74 years and over 75 years of age (Table), while it remained within the expected values for children.

The proportions of hospitalisations after ED discharge for AGE and vomiting, 8.6% (670/7,777) of people aged 15–74 years and 40.6% (479/1,180) of people 75 years of age and over, were slightly lower than during similar periods of the 7 previous years (9.9% and 44.4%, respectively).

**Figure 3:**
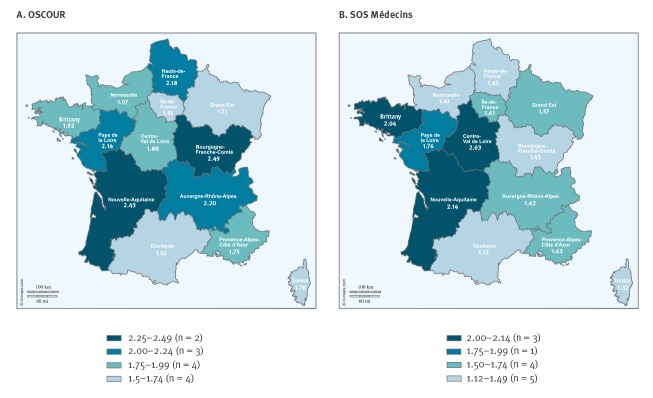
Regional distribution of the ratio between the proportion of acute gastroenteritis ED (OSCOUR) (A) and SOS Médecins (B) visits observed during the outbreak and the mean proportion calculated on the 7 previous equivalent periods (2012-2018), 26 December 2019–5 January 2020, mainland France and Corsica (n = 13)

ED: emergency department; OSCOUR: Organisation de la surveillance coordonnée des urgences; SOS Médecins: emergency GP house-call association.

All mainland France regions and Corsica were impacted by the outbreak according to the two data sources (Figure 3). Over the outbreak period, the proportion of AGE visits among total activity was more than twice as high compared with the mean proportion calculated for the same period of the 7 previous years in five of 13 regions.

## Description of notified food-borne event outbreaks suspected to be linked to raw shellfish consumption

**Figure 4:**
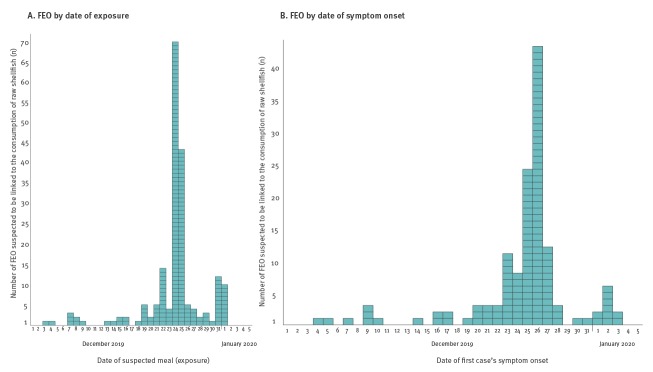
Number of food-borne event outbreaks suspected to be linked to the consumption of raw shellfish reported through the mandatory notification system, by date of suspected meal (A) and date of first case’s symptom onset (B)^a^, 1 December 2019–5 January 2020, mainland France and Corsica

In total, from 11 December 2019 to 22 January 2020, 197 FEO suspected to be linked to the consumption of raw shellfish were reported. Cases were defined as any individual presenting symptoms of gastroenteritis in the context of a FEO. The 197 reported FEO affected 1,121 persons, of which 25 (2.2%) were hospitalised. No death was reported. Cases were mostly people 15 years of age and over (96.8%; 695/719 cases with age information available). These FEO occurred in all regions of mainland France (no FEO was notified in Corsica). Dates of suspected meals ranged from 3 December 2019 to 1 January 2020 (Figure 4A), with a peak on 24 and 25 December (57.4%; 113/197). A second, smaller, peak was observed on 31 December 2019 and 1 January 2020, with 22 FEO reported (11.2%). Symptom onset ranged from 4 December 2019 to 3 January 2020, with a peak on 26 December (Figure 4B). Symptoms, mainly diarrhoea and vomiting, and incubation period were consistent with norovirus or other enteric virus infections. Epidemiological investigations undertaken by ARS and by DDecPP suspected raw shellfish, mostly oysters, as a common exposure of the cases in each of these FEO.

FEO: food-borne event outbreaks.

^a^ Information on date of suspected meal available for 195/197 FEO and information on date of symptom onset available for 137/197 FEO.

Stool samples were collected from 32 cases, corresponding to 19 FEO, and sent to the National Reference Centre (NRC) for gastroenteritis viruses. Norovirus genogroups I (GI), II (GII) or GI/GII were confirmed in 26 cases. Co-infection between norovirus and other enteric viruses was identified in 10 of 26 cases: rotavirus (n = 1), sapovirus (n = 5), enterovirus (n = 3) and Aichi virus (n = 1). Enterovirus alone was identified in two cases. A total of 35 strains, isolated from 26 cases, were characterised, and shown to belong to 10 different norovirus genotypes of both genogroups. A predominance of GI.1 (n = 10 FEO) and GII.17 (n = 6 FEO) was observed, followed by GII.4 Sydney (n = 5 FEO) and GII.8 (n = 4 FEO).

Following FEO reporting, trace back investigations were conducted by the DDecPP. Shellfish samples were collected from cases’ homes, as well as raw shellfish producers and harvest sites, and were tested for the presence of enteric viruses. Because of confirmed norovirus contamination, 31 harvest sites were closed between 21 December 2019 and 10 January 2020 by administrative decision.

From 12 December 2019 to 14 January 2020, Luxembourg, Sweden, Denmark, Switzerland, Italy and the Netherlands reported food-borne poisoning because of the consumption of raw shellfish from France or norovirus-positive controls on raw shellfish batches, mostly bivalve shellfish, from France. This information was communicated through 11 notifications in the Rapid Alert System for Food and Feed (RASFF, references 2019.4381, 2020.0039, 0056, 0059, 0091, 0100, 0130, 0133, 0139, 0186, 0187). France published three notifications in the RASFF (2020.0135, 0154, 0185) regarding the withdrawal of raw shellfish harvested in France and the possible presence of norovirus in oysters from France.

## Discussion

As the increased activity observed through the syndromic surveillance system was concomitant with a large increase in FEO suspected to be linked to the consumption of raw shellfish, the events appear to be related. Several elements suggest that FEO, in addition to typical seasonal AGE activity, contributed to the observed peak in consultations: (i) the very sudden and sharp increase of emergency medical visits for gastrointestinal illness suggests common sources of contamination compared with human-to-human transmission which would have been characterised by greater spread in the curve; (ii) the timing of the peaks during the Christmas and New Year holiday period when oysters are more commonly consumed in France; (iii) the increase primarily concerned people 15 years of age and over, individuals who are more likely to eat shellfish than children.

Furthermore, the activity for AGE and vomiting, and the number of FEO notifications decreased following the start of the new year, which coincided with the end of the holiday period and thus less oyster consumption, as well as the closure of contaminated harvest sites. Several norovirus FEO linked to raw shellfish consumption have been described in previous years during winter periods [6,7]. Concomitantly, several European countries reported food-borne poisoning and norovirus-positive controls on raw shellfish batches exported from France and originating from harvest sites that were later closed. As at 18 February 2020, two additional RASFF notifications were published by France on 22 and 24 January 2020 regarding possible contamination of norovirus in bivalve molluscs and subsequent withdrawal measures (RASFF 2020.0307 and 0362) Additionally, between 14 January and 10 February, 18 RASFF notifications were published by five European Union countries (Italy, Netherlands, Denmark, Sweden and Finland) regarding norovirus contamination of live oysters from France (RASFF 2020.0217, 0223, 0292, 0345, 0375, 0431, 0445, 0450, 0458, 0482, 0483, 0498, 0504, 0564, 0571, 0626, 0638, 0654).

No circulation of a new emerging norovirus strain or variant has been identified by the NRC that could explain the observed increase in AGE during the current winter season. As at 18 February 2020, 28% of all strains identified at the NRC were genogroup GI, the same proportion as in the previous winter season (data not shown).

As the proportions of hospitalisations after ED discharge and following a FEO were low, there is no indication of associated severe disease related to these outbreaks. A previous study estimated than 33.4% of AGE cases consulted a physician and 0.2% went to the ED [8]. Therefore, the total number of cases was probably underestimated since many persons with AGE do not consult for their mild symptoms [8].

Further research is needed in order to estimate the size of the outbreak, and to assess the proportion of cases that can be attributed to food poisoning after the consumption of raw shellfish. We plan to estimate the excess number of cases of gastrointestinal illness that occurred during Christmas and New Year period using health insurance data that will be available within 3 months.

The large amount of reported FEO suspected to be linked to the consumption of raw shellfish suggests wide-spread contamination of harvest sites. Following FEO reporting, 31 harvest sites were closed because of norovirus contamination, an unusually high number compared with previous years (11 in January 2018 and two in January 2019). Previous studies have shown that contamination of oysters by norovirus is usually linked to a combination of environmental conditions, including heavy rainfalls before harvesting [6,9]. Heavy precipitation can lead to an overflow of sewage treatment stations and then faecal contamination of the water [9,10]. During December 2019, unusually heavy rainfall was recorded across mainland France and this may have contributed to the contamination of harvest sites by human norovirus. Further study of the drivers of the wide-spread contamination of shellfish production areas must be carried out, particularly exploring precipitation data, incidents of overflow of waste water treatment plants and data on faecal contamination of the water at these production sites. Doing so will contribute to a better understanding of the variables impacting shellfish contamination and could be used by decision-makers to put early warning systems to prevent recurrence of such episodes in place.
